# Elective surgery treatment in patient living in rural area with history of recurrent primary spontaneous pneumothorax: A procedure to avoid in absence of pneumothorax. A case report

**DOI:** 10.1016/j.ijscr.2018.11.061

**Published:** 2018-11-27

**Authors:** Umberto Caterino, Davide Di Natale, Dario Amore, Roberto Scaramuzzi, Marcellino Cicalese, Pasquale Imitazione, Albina Palma, Carlo Curcio

**Affiliations:** a Thoracic Endoscopic Unit, Monaldi Hospital, Naples, Italy; b Division of Thoracic Surgery, Monaldi Hospital, Naples, Italy; c Department of Respiratory Disease, Monaldi Hospital, Naples, Italy; d Emergency Department, C.T.O., Naples, Italy

**Keywords:** Primary spontaneous pneumothorax, Recurrence, Video-assisted thoracoscopic surgery

## Abstract

**Introduction:**

Pneumothorax is defined as accumulation of air in the pleural space with secondary lung collapse resulting in dyspnea or chest pain. Currently the optimal management of spontaneous pneumothorax has been standardized, but the question of elective surgery treatment remains unresolved in patients living in rural area with history of recurrent Primary spontaneous pneumothorax [PSP].

**Presentation of case:**

A 41 years-old white man living in rural area, with a history of recurrent right spontaneous pneumothorax (three subsequent episodes) treated by positioning of chest tube, was admitted to our unit.

No respiratory symptoms and normal physical exam were observed on admission although anxiety states was noted. Chest CT scan showed small apical bullae in the right upper lobe without cystic change in the pulmonary parenchyma.

**Discussion:**

Recurrence of primary spontaneous pneumothorax represents a complication most frequently occurring within the first year.

Among the treatment options, surgical management is needed in 25–50% of all patients suffering from PSP, due to persistent air leak or recurrence.

We report the operative complications after elective surgery in a white man living in rural area with a history of recurrent right primary spontaneous pneumothorax.

**Conclusion:**

This case report highlights that elective surgery in patients living in rural area with history of recurrence PSP can lead to post-operative complications as bleeding or prolonged air leaks following the lysis of multiple pleural adhesions.

## Highlights

• Primary Spontaneus Pneumothorax is a common clinical problem.
• The risk of recurrence is high.
• The elective surgery treatment in patient with history of recurrent PSP remains questionable.
• Bleeding or prolonged air leaks represent the most common complications following the lysis of multiple pleural adhesions.
• We do not recommend the elective surgery treatment in patient living in rural area with recurrent primary spontaneous pneumothorax.

## 1 Introduction

In literature two types of spontaneus pneumothoraces has been identified according to absence or presence of underlying lung disease. Primary spontaneous pneumothorax [PSP] is more commonly diagnosed in young, thin males without underlying lung disease [[1], [2], [3]].

Secondary spontaneous pneumothorax usually occurs in patients with overt underlying lung disease, most commonly chronic obstructive pulmonary disease (COPD) and emphysema [3].

The most frequent complication of PSP is recurrence which is estimated approximately 30% with individual studies including recurrence rate of between 16 and 52% [[2], [3], [4], [5], [6], [7], [8]].

The management of PSP includes elimination of air from the pleural space and prevention of future recurrence.

Currently the optimal management of PSP, both at first pneumothorax episode and at recurrence, has been standardized, but the question of elective surgery treatment in patients living in rural area with history of recurrent PSP remains unresolved.

This work has been reported in accordance with the Scare criteria [9].

## 2 Case report

We report a case of a 41 years-old white man living in rural area with a history of recurrent right spontaneous pneumothorax (three subsequent episodes) treated with chest tube.

No respiratory symptoms and normal physical exam were observed on admission. Disability for anxiety states from fear of recurrence PSP was observed.

**Fig. 1:**
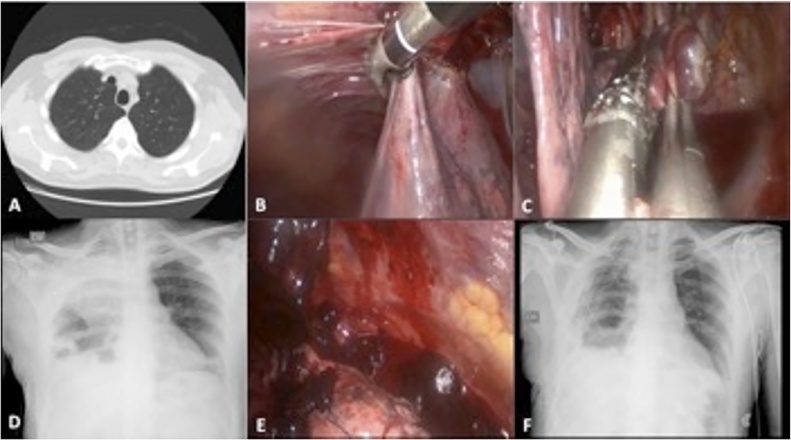
Pre operative CT-scan showed an apical bullae in the right upper lobe, in absence of pneumothorax. (A) Partial lysis of pleural adhesions and apical bullectomy were done using vessel sealing and stapling devices. (B–C). A 24 h post operative chest X-ray showed a right pulmonary radiopacity suspected for hemothorax. The presence of clots were confirmed in course of redo-VATS. (D–E) Post operative chest X-ray showed full re-expansion of the pulmonary parenchyma following to complete pleurolysis in redo-VATS. (F).

Chest CT scan showed small apical bullae in the right upper lobe without cystic change in the pulmonary parenchyma (Fig. 1A).

For prevention of recurrent PSP, we performed a bullectomy, in patient placed in the lateral decubitus position, by single-port VATS in general anesthesia with one-lung ventilation, using a 10mm-30° thoracoscope and endoscopic devices. Due to the presence of diffuse pleural adhesions, a partial pleurolysis was performed before stapling apical bullae (Fig. 1B, C).

A 24-Fr chest tube was introduced after the procedure with the closure of the access incision.

After 24 h the post operative chest X-ray revealed a right radiopacity suspected for hemothorax. (Fig. 1D) A redo-VATS, under general anesthesia with one lung ventilation, was performed by biportal anterior approach. This surgical procedure allowed to remove blood clots and residual adhesions in the pleural cavity with full expansion of the lung (Fig. 1E, F). Two 24-Fr chest tubes were placed in the pleural cavity at the end of the operation.

The patient had post-operative air leaks which resolved after 10 days and he was discharged thereafter.

## 3 Discussion

Recurrence of primary spontaneous pneumothorax represents a complication most frequently occurring within the first year.

Among the treatment options, surgical management is needed in 25–50% of all patients suffering from PSP, due to persistent air leak or recurrence [4].

The objective of surgical management of pneumothorax is twofold: firstly, to resect the visible pleural bullae or blebs responsible for the air leak; secondly, to obtain pleurodesis of parietal and visceral pleural surfaces.

Open thoracotomy with resection of bullae and pleural abrasion has been widely used in the past. Although this surgical approach is associated to lower recurrence rates, it has been replaced by video-assisted thoracoscopic surgery (VATS) thanks to better early clinical post operative outcomes such as minor blood loss, lower patient pain and shorter hospital stay [5,6].

Nowadays, VATS bullectomy and pleurodesis is widely accepted as a safe and reliable option for treatment of recurrent spontaneous pneumothorax [7].

Currently the optimal management of PSP, both at first pneumothorax episode and at recurrence, has been standardized, but the question of elective surgery treatment in patients living in rural area with history of recurrent PSP suffering from anxiety states remains unresolved.

Patients living in rural area have difficult to reach hospital emergency department.

This condition determines anxiety states if acute illness is associated with risk of increased mortality.

In our case, the anxiety states secondary to history of recurrent right primary spontaneous pneumothorax and the major risk of mortality related to acute respiratory failure and distance to hospital have influenced our management approach.

## 4 Conclusion

This case report highlights that elective surgery in patients living in rural area with history of recurrence PSP can lead to post-operative complications as bleeding or prolonged air leaks following the lysis of multiple pleural adhesions.

Finally we strongly recommend the surgical treatment of PSP recurrence at the time of acute episode.

## Conflicts of interest

The authors have no conflicts of interest to declare.

## Sources of funding

None.

## Ethical approval

This case reports not needed ethical approval.

## Consent

Written informed consent was obtained from the patient for publication of this case report.

## Author contributions

Dott. Caterino Umberto and dott Dario Amore have written the paper.

The remaining authors are contributors.

## Registration of research studies

This work is case report and there is no need of registration.

## Guarantor

Dott Caterino is guarantor for the work.

## Provenance and peer review

Not commissioned, externally peer reviewed.
